# Ancient Japanese Dental Art

**Published:** 1900-01-15

**Authors:** T. A. Long

**Affiliations:** Philadelphia, Pa.


### ANCIENT JAPANESE DENTAL ART.

BY T. A. LONG, PHILADELPHIA, PA.

Read before the Ohio State Dental Society, December, 1899.

It is but a few years since the Japanese have been brought to our notice in the arts and sciences, although they, as a nation, are very much older than we, and in the dental art antedate us by more than a century. We find examples of their skill in artificial plates, older than this nation--made before the discovery of America. In this work we find rare skill displayed, and an amount of patience expended that seems marvelous.

When we consider the crude materials and tools with which they had to work, and consider the outcome of their patient labor, we must acknowledge the bright intellect that produces such exquisite results.

There is an old story to the effect that a king, to show the great skill of his people in the arts, sent to the ruler of another country a fine cambric needle, as a sample of their workmanship. This needle was returned to the sender with an eye drilled through the point, into which was inserted another finer needle, showing the superior workmanship of the people to whom the original was sent.

We have almost a similar example before us in the collection of Japanese artificial plates on exhibition here, in a case of celluloid plate mounted with human teeth. This Japanese student, educated at one of our colleges, was taught to make celluloid plates mounted with porcelain teeth in the ordinary way. He then went back to Japan, and sent us as a specimen of his skill, a celluloid plate mounted with human teeth. It is quite doubtful whether this specimen could be duplicated in this country.

It is only within a comparatively few years that the Japanese have made much progress in the dental art; but the progress has been rapid, and an exhibit of modern Japanese dentistry would compare favorably with any American or European collection.

There are two colleges of dentistry in Tokio, the professors all being natives. Some graduated here, others in Europe and Japan. Each school is largely attended, and all their methods and appliances are modern—such as are used and taught in Europe and America.

I have here specimens of four dental journals, which are now published in the Japanese language, and to aid you in reading them, I would say that the title page is on the back cover, and you commence to read from the top downward like a column of figures, commencing at the upper right-hand corner.

We have lately received through our Japanese dealers specimens of artificial teeth which are fairly good imitations of our forms and colors, and it is only a matter of time when the Japanese will compete with the world in almost every line of manufacture.

The company which I have the honor to represent, have, after years of patient work, made a collection of specimens of Japanese artificial dentures and instruments, wood-working tools, etc., pertaining to the dental art, which I herewith show. This will give you a better idea of the rapid strides made by them, in the pursuit of your profession, than pages of history could convey.

First on the list is a wax model of a partial case for the upper jaw. You will notice that the model and impression are both of wax. They used no plaster. The operations of making an artificial plate is as follows:

After having obtained the model of hard wax, a block of wood is selected of the proper size, into which holes are made to make spaces for any teeth that remain in the mouth. (This is for a partial denture). The operator cuts away a portion of the wood to roughly approximate the shape of the model, then covers the model with a coating of thick glutinous water-color paint (usually red), then presses this model lightly against the block of wood.

Wherever the model touches the wood it leaves a portion of the paint. After removing the model, the painted portions on the block of wood are cut away, and this operation is repeated until a perfect fit is obtained.

Before finishing the carving, the model is placed in the articular, which consists of a wooden box with a sliding shelf on to which the model is placed, and to which the block is also fastened temporarily. This is to insure closure of the model and block of wood in the same position every time.

The articulator is also brought into use, after the plate is carved to a fit, to articulate the teeth when being carved into their places on the wooden plate.

The wood used in making teeth-plates is a species of plum tree, very hard and close grained, and resembles our apple-tree wood. The tree grows to about six inches in diameter.

The teeth used were of various kinds. Many human teeth were used, but ivory and stone seemed to be the most popular. Many plates are made with the teeth carved from the same block, making the plate and teeth all of one piece. The stone teeth used are made from a grayish kind of stone, somewhat resembling our soap stone, but harder, and is not a bad imitation for color of some of our dark shades of porcelain teeth. Some plates are carved in one piece in ivory, and are beautiful examples of art in that line.

We do not find any carved molars or bicuspids in the collection, except for small partial cases, the grinding surfaces being covered with little nails with large heads, like hob nails used in the soles of coarse shoes. Some of the plates shown have been worn so long that the heads of these nails have been worn entirely off.

We have also specimens of crowns and bridges made probably a hundred years ago. One, an ivory carving of front teeth attached to the natural roots by wooden pegs. Another, a single lateral upper incisor—a human tooth—set in ivory, with a large hole made through it laterally, into which cotton is packed. When the cotton is moistened it swells and presses against the adjoining teeth, and by this means is kept in place. A very ingenious device.

When we examine these artificial plates, we are at a loss to know how some of them could ever have been retained in the mouth; but they show unmistakable evidence of having been worn for years. There are no suction or air chambers to be seen, yet some of the mouths must have had no arch at all.

One thing noticeable is the closeness of the joints where the teeth are joined to the plate. This is done by carving a socket into the plate, into which the tooth fits perfectly. A hole is made laterally through these sockets, running from one side of the plate (as far as the teeth extend) to the other. Then a string is drawn through these sockets and through corresponding holes made through the teeth laterally, thus securing them in position. No trace of this string can be seen when the piece is finished, and the string seems to last as long as the rest of the piece.

BLACK TEETH.

You will see many black teeth among the collection. These are for married women, and have been made by the S. S. White Company, for many years for the Japanese market.

There is a legend to the effect that a beautiful princess, whose husband lost his life in battle, was so grieved at her loss that in order to repel the advances of the many suitors for her hand, stained her teeth black, in order to mar her beauty. She being of high rank was a model for the rest of the high born ladies in the kingdom, so far as appearance went. The custom was adopted by the high caste ladies in sympathy with the princess, and came into general use, until the original cause was lost sight of, and it came to be looked upon as a mark of beauty and aristocratic style. These black teeth were worn by married ladies only. The custom is now almost obsolete.

EXTRACTING.

No. 98 represents a hammer or mallet used in extracting teeth, is a piece of lead wrapped in cloth, and held in the palm of the hand, and is used for striking the end of the steel punches. These punches are concave on their smaller ends, forming an edge, which is placed at an angle against the neck of the tooth to be extracted, and a blow from the mallet forces it upward out of its socket.

There are also other extracting instruments. A piece of narrow iron bent upon itself like the letter U, formed over a piece of wood, and wrapped with twine. The iron loop is placed over the tooth, and a twisting motion of the hand removes the tooth. You will notice that the instruments are very short and could easily be concealed in the hand of the operator.

You have heard the fairy tale about the Japanese dentist extracting teeth with the thumb and finger, unaided by any instrument. Some tourist, who has spent six weeks in Japan, went home and wrote a history of the country, having seen a tooth extracted in that manner undoubtedly with this instrument. The Jap was too smart to let him see the instrument.

In wood-working tools you will see that the saws are all draw cut and very thin. The Japanese woodworkers work toward them in sawing and planing. This enables them to use much thinner saw blades than we do, without danger of bending or buckling them.

In the collection you will notice a lady's outfit for staining her teeth and stenciling her eyebrows and face. The various paper packages are filled with black powder, into which is dipped the brushes made of wood, after being moistened with warm water and acid. The teeth are rubbed in this way until the desired color and polish is obtained. This operation, like hair dressing, must be repeated as often as appearance would necessitate.

I will not tire you with any further description of the specimens on exhibition, but would say that with the aid of the catalogue accompanying the exhibit you will be well repaid for your time by making a careful study of the collection.

The specimens exhibited by Mr. Long were as follows:

1. Wax model and impression of upper jaw. 2. Wax model. 3 to 8 show the operation of making a wooden plate. 9. Finished plate with front teeth of stone, with nails substituted for molars. 10. Wooden plate with teeth of ivory, stained gums, and depressions in the wood to occlude with the lower molars. 11. Wooden partial plate, front teeth of ivory, and molars of stone facing and occluding surface of nails. 12. Wooden plate with human front teeth, and nails for molars. 13. Wooden plate, stone front teeth, and nails for molars. 14. Wooden plate, stone front teeth blackened, and nails for molars. 15. Partial plate of teeth, carved from one piece of wood and stained with black. 16. Full lower plate of wood and human teeth. 17. Partial plate of wood with stone teeth. 18. Lower set of wood, front teeth of stone, molars of nails. 19. Partial set of celluloid, with porcelain teeth. 20. Partial plate of rubber, with porcelain teeth. 21. Full upper set celluloid, with human teeth. 22. Partial plate of rubber, with black porcelain teeth. 23. Lower plate of rubber, with front teeth of black rubber and molars of porcelain. 24 and 25. Upper partial set, with plate and teeth of ivory, carved in one piece. 26. Piece of bridge-work of ivory, carved in one piece. 27. Single crown, a human tooth set in ivory. 28. A single tooth and partial plate, carved in ivory in one piece, with the tooth stained black. 29. Two upper centrals and small plate, carved from stone in one piece. 30. Lower partial plate, with human teeth set on ivory plate. 31. Four upper incisors, with teeth and plate of ivory, in one piece. 32. Bicuspid and molar, carved in one piece in ivory. 33 to 95. Old plates worn out and discarded, showing various methods of construction. 96 and 99. Articulators. 97. Grass used for polishing wood. 98. Hammer for extracting with a punch. 100. Ax. 101. Drill. 102. Adze. 103. Stencil for decorating the face, eyebrows, etc. 104 to 109. Steel files. 110 and 111. Shark-skin files. 112. Sea-grass file. 113 and 115. Stone files. 114. Piece of shark skin. 116 to 120. Chisels. 121 to 125. Saws. 126 to 140. Wood-working tools. 141 to 151. Extracting instruments. 152 to 161. Probes, gum lancets, scalers, etc. 162. Tongue-holder. 163. Small hammer. 164 and 166. Pliers. 165. Shears. 167 to 170. Modern Japanese forceps. 171 to 204 represent an outfit for cleaning and staining the teeth.

